# Ammonia Volatilization Loss and Corn Nitrogen Nutrition and Productivity with Efficiency Enhanced UAN and Urea under No-tillage

**DOI:** 10.1038/s41598-019-42912-5

**Published:** 2019-04-29

**Authors:** Shuangli Liu, Xiaohui Wang, Xinhua Yin, Hubert J. Savoy, Angela McClure, Michael E. Essington

**Affiliations:** 1 Associate Professor, National & Local Joint Engineering Research Center for Ginseng Breeding and Application, Jilin Agricultural University, Changchun, Jilin 130118 China; 2 Associate Professor, Research Center of Agricultural Environment and Resources, Jilin Academy of Agricultural Sciences, Changchun, Jilin 130033 China; 3 Research Specialist, Visiting Scientist, Associate Professor, and Full Professor, Department of Plant Sciences, The University of Tennessee, 605 Airways Blvd., Jackson, TN 38301 USA; 4 Associate Professor and Full Professor, Department of Biosystems Engineering and Soil Science, The University of Tennessee, 2506 E. J. Chapman Drive, Knoxville, TN 37996 USA

**Keywords:** Plant physiology, Geochemistry

## Abstract

New urease and nitrification inhibitors and polymer coatings were introduced in recent years, but their effects on N loss and plant N nutrition were scarcely examined in agronomic no-tillage production systems. A field experiment of urea treated with efficiency enhancers was conducted on no-tillage corn (*Zea mays* L.) in Tennessee, the USA during 2013–2015. A field experiment on urea and ammonium nitrate (UAN) treated with efficiency enhancers was carried out on no-tillage corn in Tennessee in 2014 and 2015. Urea treated with N-(n-butyl) thiophosphoric triamide (NBPT) at concentrations of 20% (NBPT_1_), 26.7% (NBPT_2_), or 30% (NBPT_3_) and polymer coated urea (PCU) were effective but maleic-itaconic copolymer treated urea was ineffective in reducing ammonia volatilization loss and improving N nutrition, grain yield, and N agronomic use efficiency of corn compared with untreated urea. Specifically, NBPT_1_, NBPT_2_, or NBPT_3_ treated urea and PCU reduced the total ammonia volatilization loss by 29.1–78.8%, 35.4–81.9%, 77.3–87.4%, and 59.1–83.3% during the 20 days after N applications, but increased grain yield by 15.6–31.4%, 12.9–34.8%, 18.7–19.9%, and 14.6–41.1%, respectively. The inhibitory effect of NBPT on ammonia volatilization did not improve with NBPT concentration increased from 20% to 30%. UAN treated with NBPT_3_ or a combination of urease and nitrification inhibitors resulted in 16.5–16.6% higher corn yield than untreated UAN only when they were surface applied. In conclusion, when urea-containing fertilizers are surface applied without any incorporation into the soil under no-tillage, their use efficiencies and performances on corn can be enhanced with an effective urease inhibitor in areas and years with noticeable urea N losses.

## Introduction

Nitrogen fertilizer is often applied on the soil surface in no-tillage production systems without any mechanical incorporation into the soil in many areas around the world such as the State of Tennessee^1^. Ammonium nitrate (AN) can be surface broadcast since it has a low potential for N loss to the atmosphere via ammonia volatilization^2^. However, urea-containing fertilizers such as urea and fluid urea and ammonium nitrate (UAN) have larger potential for ammonia volatilization loss when they are applied to soil surface and not incorporated into the soil by tillage or rainfall^2^. Application rate of N fertilizer, soil pH, and soil moisture are among the key factors affecting ammonia volatilization loss. Ma *et al*.^3^ reported that enhancing N application rate increased ammonia volatilization loss in corn production. Martens and Bremner^4^ found ammonia volatilization loss is positively correlated with soil pH. Ammonia volatilization loss is lower when soils are dry due to slower dissolution thus slower hydrolysis of urea^5^. As soil moisture increases, ammonia volatilization loss increases. Direct N losses of the applied N from conventional agricultural systems ranged from 10% to 78%, and on average 40% of urea N can be lost within days of application^6,7^. Ammonia volatilization loss is expected to be greater from surface applied urea without incorporation into the soil under no-tillage than surface applied urea incorporated by tillage operations under conventional tillage. Greater ammonia volatilization loss in a no-till system is also related to the fact that there are more crop residues on soil surface under no-tillage than conventional tillage, so more urease enzyme is present in no-till soils, which will result in quicker and greater ammonia volatilization loss from urea. No-tillage has become increasingly adopted around the world because of its significant benefits in soil and water conservation. Similar to many other regions, no-tillage is very widely used in Tennessee, encompassing 75% of planted corn acreage in 2016^8^. Therefore, more research is warranted to develop innovative practices that can apply urea-containing fertilizers more efficiently in no-tillage production systems.

There are several major types of urease and nitrification inhibitors and coated urea available on the market. One urease inhibitor product is N-(n-butyl) thiophosphoric triamide (NBPT). It can reduce ammonia volatilization loss and thus enhance N fertilizer use efficiency by inhibiting the activity of the urease enzyme^9,10^. As a result, there will be more fertilizer N in the soil available for the crop to take up. Agrotain [NBPT1, 20% N-(n-butyl) thiophosphoric triamide, 3.3 kg NBPT1 per 1000 kg urea], Agrotain Ultra (NBPT2, 26.7% N-(n-butyl) thiophosphoric triamide, 3.3 kg NBPT2 per 1000 kg urea), Agrotain Advanced [NBPT3, 30% N-(n-butyl) thiophosphoric triamide; 3.3 kg NBPT3 per 1000 kg urea], and Agrotain Plus [NBPTNI, 6.5% N-(n-butyl) thiophosphoric triamide +81.2% dicyandiamide; 6.80 kg NBPTNI per 1000 kg UAN] are among the key brands which use NBPT manufactured by the Koch Industries, Inc. (Wichita, KS)^11^. Another inhibitor type is a co-polymer of maleic and itaconic acids (MIC) for treating urea, UAN, and manure originally marketed as Nutrisphere by Specialty Fertilizer Products, LLC (Leawood, KS). It slows down the breakdown of urea-containing fertilizers so that urea N is captured in the soil for a longer time via reducing volatilization and nitrification losses. The third type of product is polymer coated urea (PCU). It can release N slower but for a longer time, and can be manipulated through selecting type and concentration of the coating material during manufacturing to match up with the N uptake pattern of a crop over time^12^. For example, Environmentally Smart Nitrogen (ESN) manufactured by Agrium, Inc. (Calgary, Canada) is a type of PCU which releases urea N as soil warms up.

Various studies on products purported to reduce ammonia volatilization loss from urea-containing fertilizers have been conducted^13,14^, but the majority of them were conducted under conventional tillage systems. Results showed that urea + NBPT1 most strongly reduced ammonia volatilization loss when urea is surface applied and not incorporated into the soil quickly by tillage, rainfall, or irrigation^9,10^. After a meta-analysis of N efficiency enhancement products on various crops from 28 experiments in the US and six other countries, Abalos *et al*.^15^ reported that crop yield was increased by 10% with NBPT1, and yield response to NBPT1 was greater at higher N application rates, on coarse textured soils, and under irrigation. Hendrickson^16^ found from 78 sites of field corn studies across 17 US states in five years that yield was increased by 3.8% on average for urea + NBPT1 relative to urea. However, Frame *et al*.^17^ observed no corn yield improvement with urea + NBPT1 relative to urea in North Carolina and Virginia.

Positive crop yield responses were rarely found with the addition of MIC onto urea or UAN. Cahill *et al*.^18^ reported similar corn and winter wheat (*Triticum aestivum* L.) yields with UAN + MIC relative to UAN. Franzen *et al*.^19^ observed that MIC treated N produced similar yield as untreated N in flooded rice (*Oryza sativa* L.) in Arkansas and Mississippi and spring (*Triticum aestivum* L.) and durum (*Triticum durum* Desf.) wheat in North Dakota.

Recent studies have frequently shown significant yield improvements with PCU applications^20,21^, and the beneficial effects on reduction of ammonia volatilization loss could be expected with PCU as the amount of N exposed to the soil at any one time is reduced. Gordon *et al*.^12^ reported higher corn yields for PCU than urea in Kansas. Noellsch *et al*.^22^ found in Missouri that corn yield was increased with PCU relative to urea. However, corn yields did not differ between PCU and urea in Colorado and Missouri^23,24^.

The objectives of this research were to (1) examine ammonia volatilization losses, plant growth and N nutrition, grain yield, and soil N of urea treated with efficiency enhanced products relative to urea and the traditional N fertilizer AN via surface application without incorporation for no-till corn; and (2) evaluate the effects of surface applied or knifed-in UAN treated with NBPT_3_ or NBPTNI on corn productivity under no-tillage.

## Results and Discussion

### Effects of Efficiency Enhanced Urea on Ammonia Volatilization Loss

**Figure 1:**
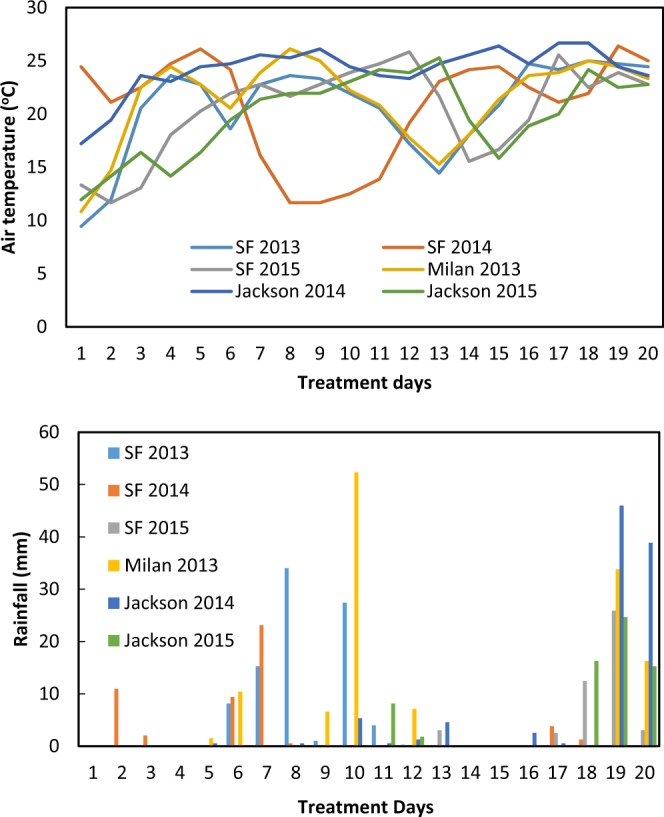
The daily air temperature and rainfall at Springfield, Milan, and Jackson during the ammonia volatilization loss measurement period of 2013 to 2015. SF: Springfield.

Treating urea with urease inhibitors or polymer coating had significant effect on total ammonia volatilization loss at all site-years except Jackson in 2014 (Table 1). In general, NBPT_1_, NBPT_2_, or NBPT_3_ treated urea and PCU resulted in lower total ammonia volatilization loss than urea + MIC and untreated urea, but had similar total volatilization loss as AN during the 20 days after N applications. Specifically, NBPT_1_, NBPT_2_, or NBPT_3_ treated urea and PCU reduced the total volatilization loss by 29.1–78.8%, 35.4–81.9%, 77.3–87.4%, and 59.1–83.3% during the 20 days after N applications. The total ammonia volatilization loss generally showed a positive relationship with air temperatures during the 20 days after N applications (Fig. 1).

**Table 1:** Effects of urea treated with urease inhibitors or polymer coating on ammonia volatilization loss at Milan, Jackson, and Springfield during 2013–2015.

Treatment	Ammonia volatilization loss (kg ha^−1^)					
	2013		2014		2015	
	Milan	SF	Jackson	SF	Jackson	SF
Zero N	0.07c	0.16b	0.13c	0.17b	0.05b	0.11b
AN	0.10c	0.60b	0.32c	0.24b	0.43b	0.28b
Urea	0.54ab	1.27a	1.23bc	2.93a	6.11a	5.30a
Urea + NBPT_1_	0.29bc	0.90b	2.87a	0.62b	1.56b	1.23b
Urea + NBPT_2_	0.24c	0.82b	2.46ab	0.53b	1.57b	1.12b
Urea + MIC	0.79a	14.21a	1.94ab	3.33a	4.65a	7.28a
PCU	0.14c	0.52b	2.03ab	0.49b	1.28b	1.23b
Urea + NBPT_3_	—	—	—	—	1.39b	0.67b
P-value	0.0040	0.0060	0.0569	0.0001	0.0013	0.0007

SF: Springfield. Means in a column within each site-year followed by the same letter are not significantly different at *P* = 0.05 according to the Fisher’s protected least significant difference (LSD). AN: Ammonium nitrate; NBPT_1_: N-(n-butyl) thiophosphoric triamide 20%; NBPT_2_: N-(n-butyl) thiophosphoric triamide 26.7%; NBPT_3_: N-(n-butyl) thiophosphoric triamide 30%; MIC: maleic-itaconic copolymer; PCU: Polymer coated urea.

Our study also showed that NBPT_1_ and NBPT_2_ efficiencies were high for the first 6 days, but then reduced after 8 days (Table 1A, Appendix). Similarly, Rawluk *et al*.^25^ observed NBPT efficiency reached to as high as 96% during the first 5 to 8 days, but throughout the last 12 to 21 days, the amount of ammonia emitted was similar for all treatments. Our result is similar to those of Mira *et al*.^26^ in that increased NBPT concentration delayed the time to reach maximum ammonia volatilization loss.

Overall, the ammonia volatilization loss was lower than our expectation at all site-years in this study, but the relative comparisons of these values are probably still rational among the treatments. The possible reason for the low ammonia volatilization loss data regardless of treatment in this study was likely related to the nature of this simple field method for measuring ammonia volatilization loss. However, this ammonia volatilization loss measuring method is commonly used in field experiments because of its simplicity (David Dunn at University of Missouri, personal communications)^3^. Ma *et al*.^3^ reported that the total ammonia volatilization loss within 28 days after N fertilization was almost all below 12 kg ha^−1^ regardless of treatment, many were less than 1 kg ha^−1^, and the lowest total ammonia volatilization loss was only 0.05 kg ha^−1^ at seven site-years^3^.

In this study, the plastic container covered over the soil prevented any rainfall during the measurement period of ammonia volatilization loss. Furthermore, the air within the plastic container was isolated from the outside and not affected by the scrubbing effect of wind. As a whole, the effects of the inverted plastic container might have significantly decreased the ammonia volatilization loss relative to that under the true field condition.

### Effects of Efficiency Enhanced Urea on Soil N Fertility

There were significant differences in ammonium N, nitrate N, and the sum of ammonium N and nitrate N in the soil among the treatments 10 days after N applications at Milan and Jackson in all years except ammonium N in 2013 (Table 2). At Jackson in 2014, soil ammonium N concentration was higher under urea + NBPT_1_ than urea or AN. At Jackson in 2015, soil ammonium N level was higher under urea + NBPT_2_ than urea. Soil nitrate N concentration was higher under AN than the other treatments in 2013 and 2015. The sum of soil ammonium N and nitrate N concentrations was higher with AN than the other treatments in 2013 and 2015 except urea + NBPT_2_ in 2013.

**Table 2:** Effects of urea treated with urease inhibitors or polymer coating on soil inorganic N in 0–15 cm 10 days after N applications at Milan and Jackson during 2013–2015.

Site-year and treatment	NH_4_^+^-N	NO_3_^−^-N	Total
	kg ha^−1^	kg ha^−1^	kg ha^−1^
**Milan-2013**			
Zero N	24.2a	13.0d	37.2d
AN	27.6a	100.0a	127.6a
Urea	25.3a	41.7c	67.0 cd
Urea + NBPT_1_	29.8a	55.6bc	85.4bc
Urea + NBPT_2_	29.8a	74.9b	104.7ab
Urea + MIC	25.1a	43.5c	68.6 cd
PCU	44.8a	38.1c	83.0bc
P-value	0.0828	<0.0001	0.0005
**Jackson-2014**			
Zero N	11.4d	21.5b	33.0b
AN	22.6 cd	93.9a	116.6a
Urea	20.4 cd	81.4a	101.6a
Urea + NBPT_1_	41.0a	100.2a	141.2a
Urea + NBPT_2_	33.4abc	103.1a	136.5a
Urea + MIC	23.1bcd	93.5a	116.6a
PCU	36.8ab	80.7a	117.5a
P-value	0.0217	0.0168	0.0097
**Jackson-2015**			
Zero N	10.3d	14.6d	24.9d
AN	42.4ab	93.5a	135.9a
Urea	29.6bc	39.5bc	69.1bc
Urea + NBPT_1_	42.6ab	40.4bc	83.0b
Urea + NBPT_2_	44.8a	45.3b	92.4b
Urea + MIC	35.9abc	48.2b	84.1b
PCU	32.3abc	39.0bc	71.3bc
Urea + NBPT_3_	35.9abc	48.2b	84.1b
P-value	0.0031	<0.0001	<0.0001

Means in a column within each site-year followed by the same letter are not significantly different at *P* = 0.05 according to the Fisher’s protected LSD. AN: Ammonium nitrate; NBPT_1_: N-(n-butyl) thiophosphoric triamide 20%; NBPT_2_: N-(n-butyl) thiophosphoric triamide 26.7%; NBPT_3_: N-(n-butyl) thiophosphoric triamide 30%; MIC: maleic-itaconic copolymer; PCU: Polymer coated urea.

In this study, soil ammonium N concentration was higher under urea + NBPT_1_ in 2014 and urea + NBPT_2_ in 2015 than urea on the 10^th^ day after application. The cumulative ammonia volatilization loss during the first six days was lower, resulting in more urea left in the soil by the 6^th^ day, which contributed to the higher soil ammonium N levels on the 10^th^ day under urea + NBPT_1_ in 2014 and urea + NBPT_2_ in 2015 than urea. These results are similar to that of Harty *et al*.^6^ who reported that urease inhibitor was effective in slowing down the urea hydrolysis process and thus decreased ammonia volatilization loss, consequently delaying the conversion of ammonia to nitrate and retaining NH_4_^+^ longer in the soil.

### Effects of Efficiency Enhanced Urea on Early Plant Growth, Plant N Concentration, and N Agronomic Use Efficiency

Plant biomass at the sixth leaf stage (V6) did not differ among the treatments at Milan or Jackson in any year (data not presented). Since seedling corn plants grow slowly and require only a small amount of N to sustain growth before V6, and soil indigenous N can often meet the N needs of young corn plants. Similar results were obtained in the study of Zhang *et al*.^27^, who reported that plant biomass did not differ between urea + NBPT1 and urea in early sampling.

Nitrogen concentrations in plant biomass at V6 and in the leaf 30 and 60 days after N applications were higher with NBPT_1_ and NBPT_2_ treated urea than under urea, but were lower than those under AN at Milan in 2013 (Table 3). However, N concentrations in biomass and in the leaf with PCU were similar as those under AN at Milan in 2013. There was no difference in biomass N concentration or leaf N concentrations among the N applied treatments, but they all had higher biomass and leaf N levels than zero N in 2014. At Jackson in 2015, NBPT_1_ and NBPT_2_ treated urea both resulted in higher N concentrations in biomass than urea + MIC and urea. However, AN had the highest N concentrations in biomass and in leaf at 30 days than the other treatments at Jackson in 2015. Plant biomass N uptake at V6 did not differ among the N applied treatments regardless of site-year (Table 3).

**Table 3:** Effects of urea treated with urease inhibitors or polymer coating on plant N concentrations and uptake at Milan and Jackson during 2013–2015.

Site-year and treatment	Biomass N concentration at V6	Leaf N concentration 30 DAA	Leaf N concentration 60 DAA	Biomass N uptake at V6
	g kg^−1^	g kg^−1^	g kg^−1^	kg ha^−1^
**Milan-2013**				
Zero N	22.3e	20.9d	13.6d	8.9b
AN	41.0a	39.3a	24.4a	19.9a
Urea	29.6d	25.7c	16.0c	22.2a
Urea + NBPT_1_	37.0bc	30.1b	18.9b	22.2a
Urea + NBPT_2_	36.5bc	31.4b	19.4b	18.4a
Urea + MIC	34.7c	28.9bc	16.4c	16.4ab
PCU	38.8ab	36.5a	23.0a	20.6a
P-value	<0.0001	<0.0001	<0.0001	0.0210
**Jackson-2014**				
Zero N	33.6b	19.9b	16.4c	15.3b
AN	42.6a	28.4a	23.4a	21.6a
Urea	41.6a	26.0a	18.4bc	23.5a
Urea + NBPT_1_	42.1a	27.2a	20.8ab	22.5a
Urea + NBPT_2_	42.7a	25.8a	20.1b	21.1a
Urea + MIC	42.1a	25.8a	19.5bc	22.7a
PCU	38.3ab	26.2a	21.0ab	20.2a
P-value	0.0095	0.0455	0.0073	0.0283
**Jackson-2015**				
Zero N	31.5d	30.1c	16.2e	4.0b
AN	46.9a	44.4a	27.0a	10.8a
Urea	44.4c	41.1b	20.2 cd	8.7a
Urea + NBPT_1_	46.3ab	42.2b	25.1ab	11.0a
Urea + NBPT_2_	46.0ab	40.7b	22.8bc	9.9a
Urea + MIC	44.3c	41.0b	19.7d	10.8a
PCU	45.4bc	41.4b	25.5ab	10.8a
Urea + NBPT_3_	45.7abc	42.3b	22.0 cd	10.2a
P-value	<0.0001	<0.0001	<0.0001	0.0046

DAA: Days after application of N fertilizer. Means in a column within each site-year followed by the same letter are not significantly different at *P* = 0.05 according to the Fisher’s protected LSD. AN: Ammonium nitrate; NBPT_1_: N-(n-butyl) thiophosphoric triamide 20%; NBPT_2_: N-(n-butyl) thiophosphoric triamide 26.7%; NBPT_3_: N-(n-butyl) thiophosphoric triamide 30%; MIC: maleic-itaconic copolymer; PCU: Polymer coated urea.

Grain N concentrations were lower with urea treated with NBPT_2_, NBPT_3_, or MIC and untreated urea than those with AN at all site-years (Table 2A, Appendix). Urea treated with NBPT_1_ or NBPT_2_ and PCU resulted in greater grain N removal and N agronomic use efficiency than urea, but lower N removal and N agronomic use efficiency than AN regardless of site-year (Table 2A, Appendix). Previous investigations have shown that NBPT1 can increase the N agronomic use efficiency of urea applied to corn^16,28,29^.

### Effects of Efficiency Enhanced Urea on Grain Yield and Moisture

The interaction between N application rate and N source was not significant on grain yield (Table 4). At both sites in 2013, urea treated with NBPT_1_ or NBPT_2_ and PCU yielded higher than urea but lower than AN averaged over the 123 and 168 kg N ha^−1^ rates. Urea + NBPT_2_ produced similar yield as urea + NBPT_1_. Urea + MIC produced similar yield as untreated urea, but lower yield than NBPT_1_ or NBPT_2_ treated urea and PCU. The trends in 2014 and 2015 were exactly the same as those observed in 2013 except Springfield in 2014. Urea + NBPT_3_ produced similar yield as NBPT_1_ or NBPT_2_ treated urea in 2015. Yields for untreated urea were consistently greater than the zero N control at all site-years showing all the fields used in this study were N deficient. Specifically, NBPT_1_, NBPT_2_, or NBPT_3_ treated urea and PCU increased grain yield by 15.6–31.4%, 12.9–34.8%, 18.7–19.9%, and 14.6–41.1% at harvest. Although the temperatures and rainfall varied among the site-years, they were generally within the normal ranges for corn production (Fig. 1A, Appendix); which allowed corn to produce normal yields regardless of site-year.

**Table 4:** Effects of urea treated with urea inhibitors or polymer coating on corn grain yield and moisture at Milan, Jackson, and Springfield during 2013–2015.

Treatment	Yield (Mg ha^−1^)					
	2013		2014		2015	
	Milan	SF	Jackson	SF	Jackson	SF
**N source**						
Zero N	5.55e	7.14e	6.01d	10.85c	5.39e	6.52d
AN	12.38a	12.92a	12.99a	13.29ab	12.68a	12.79a
Urea	7.91d	8.98d	9.22c	13.98a	8.95d	9.03c
Urea + NBPT_1_	9.98c	11.80ab	10.66b	14.04a	11.0bc	10.78b
Urea + NBPT_2_	10.66c	11.21bc	10.41b	13.61ab	10.76c	10.22b
Urea + MIC	8.43d	10.07 cd	9.56c	13.23b	9.56d	8.97c
PCU	11.16b	11.26bc	10.95b	12.41ab	11.54b	10.35b
Urea + NBPT_3_	—	—	—	—	10.73c	10.72b
**N rate (kg N ha** ^**−1**^ **)**						
123	8.10a	10.08a	9.63a	13.02a	9.46b	9.34a
168	9.66a	10.89a	10.31a	13.08a	10.69a	10.50a
**P value**						
N source	0.0001	0.0001	0.0001	0.002	0.0001	0.0001
N rate	0.1380	0.0938	0.1433	0.8343	0.0080	0.0934
N rate × N source	0.3883	0.2227	0.1888	0.8351	0.4229	0.0513

SF: Springfield. Part of the yield data were cited from a University of Tennessee extension paper (Savoy *et al*.^47^). Means in a column within the treatments of N sources or N rates followed by the same letter are not significantly different at *P* = 0.05 according to the Fisher's protected LSD. AN: Ammonium nitrate; NBPT_1_: N-(n-butyl) thiophosphoric triamide 20%; NBPT_2_: N-(n-butyl) thiophosphoric triamide 26.7%; NBPT_3_: N-(n-butyl) thiophosphoric triamide 30%; MIC: maleic-itaconic copolymer; PCU: Polymer coated urea.

Urea treated with NBPT_1_, NBPT_2_, or MIC and PCU usually resulted in lower grain moisture content than AN but similar or higher moisture level than urea averaged over the two N rates at Milan and Jackson during the study years (Table 3A, Appendix). However, there was almost no difference in grain moisture among the N applied treatments at Springfield in any year.

Higher corn yield under urea treated with NBPT1 and PCU than untreated urea in this study is in agreement with those of many other studies. Gordon^12^ showed that treated urea fertilizer yielded greater in corn than urea. Silva *et al*.^30^ reported that yield increases averaged 5.3% for most crops and soils when NBPT1 was applied. The main explanation might be due to the fact that NBPT1 as a urease inhibitor can compete for the active sites of urease enzyme, slowing down urea hydrolysis. The inhibitory effect did not improve when the NBPT concentration was increased from 20% to 30% in this study. Therefore, the use of NBPT at the 20% concentration will likely generate higher economic returns due to the lower cost on the product.

Our results generally showed that corn yield, grain N removal, and N agronomic use efficiency with urea + NBPT1, NBPT2, or NBPT3 were lower than AN. This might be explained by much higher ammonia volatilization loss from urea than AN^31^. Forrestal *et al*.^32^ found that average ammonia volatilization loss from calcium ammonium nitrate was 85% lower than urea, and urea + NBPT1 caused a 78.5% reduction compared with urea.

MIC was consistently ineffective in this study. Similar results were reported by Franzen *et al*.^19^ and Goos^33^, who found that MIC has no ammonia volatilization or nitrification inhibiting properties, and spring wheat or rice did not benefit from the addition of MIC onto urea. Literature reported that MIC inhibited urease activity by complexing nickel ions within the urease enzyme and inhibited nitrification through complexing soil copper ions^34,35^. Therefore, the possible explanation for ineffective MIC might be due to the fact that MIC somehow did not complex enough Ni and Cu ions or there were too many Ni and Cu ions in the soil in this study. Chien *et al*.^36^ concluded in a literature review that the contents of active ingredients in the MIC product were not adequate for reducing ammonia volatilization loss or increasing crop yield.

Zhou *et al*.^37^ conducted an economic analysis on the data of this study with the exclusions of Springfield in 2014 and urea + NBPT_3_ in 2015, and found that urea treated with NBPT_1_ or NBPT_2_ and PCU produced higher net economic returns than urea, but this net return was significantly below those of AN under no-tillage corn production systems. If the cost was set as $1.19 kg^−1^ N for urea + NBPT1, $1.05 kg^−1^ N for urea, $0.16 kg^−1^ for corn grain, and N application rate of 168 kg ha^−1 ^^37^, then a minimum of 147 kg ha^−1^ yield gain was needed for break-even.

The effects of treated urea on ammonia volatilization loss and crop yield seemed to be more significant under no-tillage than conventional tillage. Our results from no-till corn generally agree with those of previous no-till studies. For instance, Scharf *et al*.^38^ reported that urea + NBPT1 significantly increased no-till corn and wheat yields relative to urea with surface broadcast of N fertilizers over a three-year study in Missouri, an adjacent state to the west of Tennessee. Moro *et al*.^39^ observed in Oregon that urea + NBPT1 significantly reduced ammonia volatilization loss by 39 to 53% compared with urea when the N fertilizers were broadcast on soil surface without incorporation. On the contrary, however, Kaur *et al*.^40^ reported that urea + NBPT1 did not increase corn yield relative to urea in Missouri when surfaced broadcast N was incorporated immediately after application by conventional tillage operations.

The effects of urease inhibitor treated urea and PCU on corn yield relative to urea and AN were not affected by N application rate. This result suggests that use of urea + NBPT or PCU at 168 kg N ha^−1^ which is on the lower side of the commonly used N application rates for corn by local growers in the region will still be beneficial for corn yield improvement.

### Effects of Efficiency Enhanced UAN on Leaf Chlorophyll and Grain Yield and Moisture

The effects of N source on leaf chlorophyll (SPAD, Soil and Plant Analysis Development) and grain yield and moisture were all significant in both 2014 and 2015 (Table 5). NBPTNI or NBPT_3_ treated UAN and untreated UAN either knifed in or surface applied resulted in higher leaf SPAD at the tasseling growth stage, corn yield, and moisture than the zero N check averaged over the two N rates. UAN + NBPTNI knifed in produced similar yield as UAN knifed in averaged over the two N rates in both years. However, UAN treated with NBPTNI or NBPT_3_ surface applied resulted in higher yield than UAN surface applied but still produced lower yield than untreated UAN knifed in averaged over the two N rates. Specifically, UAN treated with NBPTNI or NBPT_3_ surface applied increased yield by 16.5% and 16.6% than UAN surface applied, respectively.

**Table 5:** Effects of UAN treated with urease and nitrification inhibitors on leaf chlorophyll (SPAD) at the tasseling stage and grain yield and moisture of corn at Jackson during 2014–2015.

Treatment	SPAD		Yield (Mg ha^−1^)		Moisture (g kg^−1^)	
	2014	2015	2014	2015	2014	2015
**N source**						
Zero N	39.1b	34.6c	5.35b	4.75d	147b	159d
UAN − knifed in	51.1a	53.8a	11.27a	12.21a	160a	165a
UAN + NBPTNI − knifed in	50.4a	53.9a	11.84a	12.39a	157a	164ab
UAN − surface applied	—	50.9b		9.56c	—	162c
UAN + NBPTNI − surface applied	—	53.1a	—	11.14b	—	162c
UAN + NBPT_3_ − surface applied	—	52.8a	—	11.15b	—	163bc
**N rate**						
150 kg N ha^−1^	47.2a	49.1b	8.51b	9.72b	152a	162b
200 kg N ha^−1^	46.5a	50.6a	10.46a	10.67a	157a	164a
**P value**						
N source	0.0001	0.0001	0.0001	0.0001	0.0298	0.0001
N rate	0.3959	0.0011	0.0007	0.0338	0.0201	0.0276
N rate × N source	0.8822	0.1602	0.0676	0.4636	0.1911	0.0968

Means in a column within N sources or N rates followed by the same letter are not significantly different at *P* = 0.05 according to the Fisher’s protected LSD. NBPTNI: N-(n-butyl) thiophosphoric triamide 20% + dicyandiamide 81%; NBPT_3_: N-(n-butyl) thiophosphoric triamide 30%.

The effects of NBPTNI or NBPT_3_ treated UAN on corn yield compared with UAN were not influenced by N application rate (Table 5). This result suggests that NBPTNI or NBPT_3_ treated UAN at either a common N application rate (150 kg N ha^−1^) or a higher rate (200 kg N ha^−1^) are both beneficial for corn yield.

The knifed in treatments were applied to both sides of each corn row, while the surface applied treatments were all applied on the soil surface in a stream in the middle between rows. The lower UAN concentration due to two knifed in slots, the shorter distance of UAN from the corn row, and the knifed in of UAN in 9 cm deep might have all likely contributed to the lower anomia volatilization loss and thus insignificant corn yield response to the addition of NBPTNI or NBPT_3_ to UAN when it was knifed in. Placing N fertilizer below the soil surface reduces ammonia volatilization losses and increases fertilizer use efficiency^41^.

Although this study was conducted on no-till corn in Tennessee, the results of this study could be useful for corn grown in the southern USA with similar management practices and climate conditions. Furthermore, our results also indicated that the yield and seed quality responses of cotton, another major crop in the southern USA, to urea and UAN treated with effective efficiency enhancement products such as NBPT via surface application without incorporation likely would be significant and positive under no-tillage^42,43^.

## Conclusions

This study evaluated the effects of efficiency enhanced urea and UAN on ammonia volatilization loss and corn N nutrition and productivity under no-tillage. The results showed that urea treated with NBPT_1_, NBPT_2_, or NBPT_3_ and PCU frequently resulted in less ammonia volatilization loss but higher corn yield, grain N removal, and N agronomic use efficiency than urea. Specifically, NBPT_1_, NBPT_2_, or NBPT_3_ treated urea and PCU reduced the total ammonia volatilization loss by 29.1–78.8%, 35.4–81.9%, 77.3–87.4%, and 59.1–83.3% during the 20 days after N applications, but increased corn yield by 15.6–31.4%, 12.9–34.8%, 18.7–19.9%, and 14.6–41.1%, respectively. There was no significant difference in the aforementioned measurements among urea treated with different NBPT concentrations ranging from 20% to 30%. However, AN was more effective than urea treated with urease inhibitors and PCU in no-till corn production. Urea + MIC did not play any role in reducing ammonia volatilization or increasing corn yield. Corn yield was higher with UAN knifed in than surface applied with or without inhibitor. Treating UAN with NBPT_3_ or NBPTNI increased corn yield by 16.5% to 16.6% when UAN was surface applied, but did not improve yield when UAN was knifed into the soil. Our results suggest that NBPT is beneficial when added to urea that is surface applied to soils under no-tillage with crop residue cover under conditions of adequate moisture to promote urea hydrolysis and ammonia volatilization but without sufficient precipitation to move the urea into the soil; NBPT and PCU provides two effective alternatives to excessive rates of surface applied urea that are presently used to ensure that N will not limit corn yield. Such information is useful to corn growers who need to decide which N efficiency enhancement product should be added onto urea and UAN in corn production under no-tillage management.

## Materials and Methods

### Urea Experiment

#### Field Conditions

A field experiment was conducted on corn to examine the impacts of urea treated with efficiency enhancement products under no-tillage at the University of Tennessee’s research and education centers at Milan and Springfield in 2013 and at Jackson and Springfield in 2014 and 2015. The soil type was Loring/Henry silt loam (fine-silty, mixed, active, thermic oxyaquic Fragiudalfs/coarse-silty, mixed, active, thermic typic Fragiaqualfs) at Milan and Hamblen silt loam (fine-loamy, siliceous, semiactive, thermic fluvaquentic Eutrudepts) at Springfield in 2013, Memphis/Loring silt loam (fine-silty, mixed, active, thermic typic Hapludalfs/fine-silty, mixed, active, thermic oxyaquic Fragiudalfs) at Jackson and Staser silt loam (fine-loamy, mixed, active, thermic cumulic Hapludolls) at Springfield in 2014, and Memphis silt loam (fine-silty, mixed, active, thermic typic Hapludalfs) at Jackson and Hamblen silt loam (fine-loamy, siliceous, semiactive, thermic fluvaquentic Eutrudepts) at Springfield in 2015.

An initial composite soil sample was collected at the 0–15 cm depth from each plot at Milan on May 9, 2013 and at Jackson on May 7, 2014 and April 21, 2015. Ten probes of 2.5-cm diameter were randomly collected for each sample. After the soil samples were air dried, ground to pass through a 2-mm sieve, and thoroughly mixed, they were analyzed by the Brookside Laboratories Inc. (New Bremen, OH) with Mehlich 3 for P and K extraction and the 1 M KCl cadmium reduction method for the determination of NH_4_^+^-N and NO_3_^−^-N concentrations^44^. The initial soil testing results at Milan and Jackson with the Mehlich 3 extractant are listed in Table 6. Similarly a composite soil sample was taken from the entire test area at Springfield each year. The initial soil testing results at Springfield with the Mehlich 1 extractant are shown in Table 6.

**Table 6:** Initial soil properties prior to experiment for each site-year of the urea and UAN experiments. OM, organic matter; ND, not determined.

Experiment	Site	Year	pH	OM	NH_4_^+^-N	NO_3_^−^-N	P	K
				g kg^−1^	mg kg^−1^	mg kg^−1^	mg kg^−1^	mg kg^−1^
Urea	Milan	2013	6.7	15.5	8.8	4.2	55.5	174.8
	Jackson	2014	6.5	16.1	11.8	8.5	26.4	140.9
	Jackson	2015	6.8	12.1	4.3	4.1	30.1	147.5
	Springfield	2013	6.2	ND	ND	ND	19.5	59.5
	Springfield	2014	6.4	ND	ND	ND	19.0	87.0
	Springfield	2015	6.8	ND	ND	ND	20.5	54.5
UAN	Jackson	2014	6.6	17.8	3.5	3.9	41.0	161.0
	Jackson	2015	6.7	23.0	4.0	3.0	69.0	104.0

#### Experiment Design and Implementation

The experiment at all site-years was set up in a randomized complete block split plot design with four replications. The two N application rates of 123 kg N ha^−1^ (110 lb N a^−1^) and 168 kg N ha^−1^ (150 lb N a^−1^) were used as the main treatments, and the following N sources were used as the sub treatments: 0 kg N ha^−1^ as the control, ammonium nitrate (AN), urea, urea + N-(n-butyl) thiophosphoric triamide 20% (NBPT_1_), urea + N-(n-butyl) thiophosphoric triamide 26.7% (NBPT_2_), urea + Ca slat of maleic-itaconic copolymer 30–40% (MIC), and semipermeable polymer coated urea (PCU, 44-0-0). There was another sub treatment of urea + N-(n-butyl) thiophosphoric triamide 30% (NBPT_3_) added to the experiment in 2015 only. For the treatments of urea treated with NBPT products, 2.84 liters of liquid NBPT1, NBPT2, and NBPT3 were used to treat one ton of urea, respectively. For the urea + MIC treatment, 1.89 liters of MIC liquid product were added to one ton of urea. All these doses were based on the product use instructions from the manufacturers. Untreated urea and ammonium nitrate were included as two standard comparisons. The N rate of 123 kg N ha^−1^ was used to create a N deficiency condition for corn, and the higher 168 kg N ha^−1^ rate represents the University of Tennessee recommended N rate for a corn yield goal of 7.84 to 9.42 Mg ha^−1^ (125 to 150 bushels a^−1^). The 168 kg N ha^−1^ rate is on the lower side of the commonly used N application rates for corn by local growers in the region.

The plot size was 3 m wide and 9.1 m long with four rows of corn for all site-years. Phosphorus and K fertilizers were applied as needed based on soil testing results for each site-year before corn planting. An agricultural limestone (CaCO_3_) was applied at 560 kg ha^−1^ to the whole test area only at the Springfield site in each year. Corn cultivar DKC63-84, Wyffels 7886 RIB, DKC63-87, DKC63-25RIB, DKC63-87, and Wyffels W7736 RIB was no-till planted in 76-cm rows at a seeding rate of 79,000 plants ha^−1^ on May 2 and May 2 of 2013, May 7 and May 5 of 2014, and April 29 and April 28 of 2015 at Milan and Springfield in 2013, Jackson and Springfield in 2014, and Jackson and Springfield in 2015, respectively. Corn was grown with the standard non-irrigated no-till corn management practices of the region.

All the N treatments were implemented on May 13, May 19, and April 29 at Milan/Jackson and May 13, May 6, and April 28 at Springfield in 2013, 2014, and 2015, respectively. The treated and untreated N fertilizers were broadcast applied to soil surface of the designated plots by hand without any incorporation in the soil at the first-leaf stage (V1). Except the aforemented treatments, no additional N fertilizer was applied in any treatment at any of the site-years.

#### Data Collection

Ammonia volatilization loss was determined with the method of Ma *et al*.^3^. Immediately after the treatments were applied, 0.1 N sulfuric acid solution of 60 mL held in an uncovered jar (7.94 cm in height and 6.67 cm in diameter) was laid on the soil surface in the center area of a plot, and covered with a plastic container (36 cm high and 29 cm for the inner diameter) on a plot basis at both Milan and Springfield on May 13, 2013, at Jackson on May 19 and at Springfield on May 9 in 2014, and at Jackson on April 29 and at Springfield on April 30 in 2015. There was no opening in the plastic container that covered the soil to enable air circulation/mixing. Jars were covered with plastic containers which were placed in a 1.91 cm deep trench and secured with a heavy brick. Plastic containers were placed midway between corn rows on flat ground. The acid solution was replaced at 2, 4, 6, 8, 10, and 20 days after the N treatments, respectively, in order to measure soil ammonia volatilization loss. Sample jars were sealed with lids and brought to the Soil Chemistry Lab at the University of Tennessee, Knoxville for the determination of ammonia concentration in the acid solutions. Ammonia in the sulfuric acid solutions was analyzed using the modified Berthelot reaction method (similar to EPA method 350.1). In this procedure, ammonia reacts with alkaline phenol and hypochlorite to form indophenol blue. The color of the complex is then intensified with sodium nitroprusside. The analysis is performed using the Skalar model SAN++ automatic spectrophotometer system at a wavelength of 660 nm.

A composite soil sample per plot consisting of 10 cores was taken in 0–15 cm at Milan on May 23, 2013 and at Jackson on May 29, 2014 and May 9, 2015, 10 days after N applications. The sample was taken from the two center rows of the plot approximately 23 cm away from the row. These samples were processed and analyzed for NH_4_^+^-N and NO_3_^−^-N concentrations with the same method as used for the initial soil samples.

An aboveground plant biomass sample was collected at the sixth leaf stage (V6) at Milan on June 7, 2013 and at Jackson on June 6, 2014 and May 22, 2015 by cutting 10 plants randomly from the center two rows of each plot. These samples were dried in a forced-air oven at 65 °C till completely dry for the determination of dry biomass weight. Tissue N concentration was estimated from a composite leaf sample consisting of 10 youngest fully developed leaves, which was randomly collected from 10 plants in each plot at Milan on June 12 and July 12, 2013 and at Jackson on June 18 and July 18, 2014 and May 29 and June 29, 2015, representing 30 and 60 days after implementation of the N treatments. Leaf samples were dried with the identical method as that used for the plant biomass samples. Both plant biomass and leaf samples were ground in a Wiley mill (Arthur K. Thomas Co. Philadelphia, PA) to pass through a 1-mm screen. Total N concentrations in these samples were determined using the dry combustion method with a Leco TruSpec C and N Analyzer (Leco Corporation, St. Joseph, MI).

Grain yield was determined by harvesting the two center rows of corn of each plot using a plot combine with an automatic weighing scale and a moisture meter at Milan on September 24 and Springfield on October 1 in 2013, at Jackson on September 25 and Springfield on September 24 in 2014, and at Jackson on September 4 and Springfield on September 24 in 2015. Grain yields were adjusted for the removal of the plants per plot for plant biomass and to a standard moisture content of 155 g kg^−1^. A grain sample was collected at harvest from each plot at both sites for grain N concentration, which was determined with the same method as that for the leaf samples. Nitrogen agronomic use efficiency was calculated for each plot as follows: N agronomic use efficiency = (N removed by grain in a plot with “X” treatment − N removed by grain in a zero N plot) ÷ N applied to the plot with “X” treatment as fertilizer^45^.

Ammonia volatilization loss was measured on a plot basis from the sub treatments at the main treatment of 123 kg N ha^−1^ only due to resource restriction at all site-years; soil NH_4_^+^-N and NO_3_^−^-N concentrations, plant biomass weight, and N concentrations in leaf and plant biomass were measured for each plot from the sub treatments at the main treatment of 123 kg N ha^−1^ at Milan and Jackson only. Only grain yield and moisture were determined from all the plots regardless of treatment, site, and year.

#### Statistical Analyses

Analysis of variance was conducted with the Proc Mixed Model in SAS version 9.4 (SAS Institute, Cary, NC) for each site-year separately because the treatments were not exactly the same across all site-years. For grain yield and moisture measurements, the N application rates and N sources were treated as the main and sub treatments, respectively, under a split plot design, and both factors and their interaction were handled as the fixed experimental factors; while the replicates were treated as a random factor in both experiments. For all other measurements, the N sources were treated as the fixed experimental factor under a randomized complete block design, while the replicates were treated as a random factor. Treatment means were separated with the Fisher’s protected least significant difference (LSD). Probability values less than 0.05 were designated as statistically significant for all analyses.

### UAN Experiment

#### Field Conditions

A field experiment was carried out on corn to assess the effects of fluid N fertilizer UAN treated with efficiency enhancers at Jackson, TN in 2014 and 2015. The soil was a Loring silt loam (fine-silty, mixed, active, thermic oxyaquic Fragiudalfs) (upland) in 2014 and a Grenada silt loam (fine-silty, mixed, active, thermic oxyaquic Fraglossudalfs) (upland) in 2015. An initial composite soil sample was collected at the 0–15 cm depth across the experimental site on April 4, 2014 and March 23, 2015. The initial soil testing results with Mehlich 3 as the extractant are presented in Table 6.

#### Experiment Design and Implementation

The experiment was arranged in a randomized complete block split plot design with four replicates. The two N application rates, optimal rate (200 kg N ha^−1^) and 75% of optimal rate (150 kg N ha^−1^), were assigned to the main plots. The UAN sources: UAN − knifed in, UAN + NBPTNI − knifed in, and zero N as the check were assigned to the sub plots in 2014, and UAN − knifed in, UAN + NBPTNI − knifed in, UAN − surface applied, UAN + NBPTNI − surface applied, UAN + NBPT_3_ − surface applied, and Zero N as the check were used as the sub treatments in 2015. For the UAN + NBPTNI treatments in both years, 6.81 kilograms of dry NBPTNI product were used to treat one ton of UAN when the main treatment was 150 kg N ha^−1^, but only 6.05 kilograms being used in the main treatment of 200 kg N ha^−1^. For the urea + NBPT_3_ treatment in 2015, 1.42 liters of NBPT_3_ liquid product were used to treat one ton of UAN. All these doses were based on the product use instructions from the manufacturer.

Phosphorus and K fertilizers were applied as needed based on soil testing results for each site-year before corn planting. The plot size was 3 m × 9.1 m with four rows of corn. Corn cultivar DKC63-87 was no-till planted in 76-cm rows at a seeding rate of 79,000 plants ha^−1^ on May 7 in 2014 and 84,000 plants ha^−1^ on April 13 in 2015.

Both main and sub treatments were imposed on May 28, 2014 and May 7, 2015. The knifed in treatments of UAN + NBPTNI and UAN were applied to both sides of each corn row approximately 19 cm away from the row at an approximately 9 cm depth in the designated plots with a 4-row 3-point hitch mounted UAN applicator (The KBH Corporation, Clarksdale, MS). The surface applied treatments of UAN + NBPTNI, UAN + NBPT_3_, and UAN were all applied on the soil surface by a stream in the middle between rows using the 0015 straight stream nozzles manufactured by the Tee-Jet Technologies in Glendale Heights, IL, which were mounted on a 4-row tractor mounted spray boom.

#### Data Collection

Soil plant analysis development (SPAD) readings were taken with a Minolta SPAD-502 meter (Minolta Crop., Osaka, Japan) on the ear leaf at the tasseling stage from each split-plot in each year^46^. Measurements were taken at a central position on the leaf blade and the midrib was avoided. Twenty leaves were tested randomly in the two center rows of each split-plot and averaged to a single SPAD value. Grain yield and moisture were determined from all the split-plots on September 25, 2014 and September 4, 2015 with the same method as used in the urea experiment.

#### Statistical Analyses

Analysis of variance was conducted with the Proc Mixed Model in SAS version 9.4 (SAS Institute, Cary, NC) for each year separately because the treatments were not identical for the two years. For each measurement, the N application rates and N sources were treated as the main and sub treatments, respectively, under a split plot design, and both treatment factors and their interaction were handled as the fixed experimental factors; while the replicates were treated as a random factor. Treatment means were separated with the Fisher’s protected least significant difference (LSD). Probability values less than 0.05 were designated as statistically significant for all analyses.

## Supplementary information

At present, this entire section including Acknowledgments, Author Contributions, Competing Interestsare not well organized in terms of font type, size, bold, etc. compared to recently published articles in Scientific Reports. So pleasemake the whole section here including Acknowledgements, Author Contributions, Competing Insterests in the correct font type, size,bold, etc.
