# Supplementary material for: Ammonia Volatilization Loss and Corn Nitrogen Nutrition and Productivity with Efficiency Enhanced UAN and Urea under No-tillage
Source: Sci Rep. 2019 Apr 29;9:6610. doi: 10.1038/s41598-019-42912-5 (PMC6488641; doi:10.1038/s41598-019-42912-5)
Supplement: Supplementary file 1 — At present, this entire section including Acknowledgments, Author Contributions, Competing Interestsare not well organized in terms of font type, size, bold, etc. compared to recently published articles in Scientific Reports. So pleasemake the whole section here including Acknowledgements, Author Contributions, Competing Insterests in the correct font type, size,bold, etc. [file 41598_2019_42912_MOESM1_ESM.docx]

**Ammonia Volatilization Loss and Corn Nitrogen Nutrition and Productivity with Efficiency Enhanced UAN and Urea under No-tillage**

Shuangli Liu^a,c^, Xiaohui Wang^b,c^, Xinhua Yin^c,^*, Hubert J. Savoy^d^, Angela McClure^c^, and Michael E. Essington^d^

^a^ Assistant Professor, College of Chinese Medicinal Materials, Jilin Agricultural University, Changchun, Jilin 130118, China

^b^ Associate Professor, Research Center of Agricultural Environment and Resources, Jilin Academy of Agricultural Sciences, Changchun, Jilin 130033, China

^c^ Research Specialist, Visiting Scientist, Associate Professor, and Full Professor, Department of Plant Sciences, The University of Tennessee, 605 Airways Blvd., Jackson, TN 38301, USA

^d^ Associate Professor and Full Professor, Department of Biosystems Engineering and Soil Science, The University of Tennessee, 2506 E. J. Chapman Drive, Knoxville, TN 37996, USA

* Corresponding author: Email: xyin2@utk.edu; Phone: 1-731-425-4750; Fax: 1-731-425-4760; Address: 605 Airways Blvd., Jackson, TN 38301, USA

| **Site-year** | **Treatment** | **2 d** | **4 d** | **6 d** | **8 d** | **10 d** | **20 d** |
| --- | --- | --- | --- | --- | --- | --- | --- |
|  |  | **g N ha^-1^** | **g N ha^-1^** | **g N ha^-1^** | **g N ha^-1^** | **g N ha^-1^** | **g N ha^-1^** |
|  |  |  |  |  |  |  |  |
| Milan | Zero N | 565.6b | 44.6a | 35.4c | 15.0c | 64.0a | -- |
| -2013 | AN | 127.2b | 542.8a | 200.3c | 31.8c | 83.3a | -- |
|  | Urea | 2555.0a | 788.1a | 1807.0b | 122.1bc | 149.6a | -- |
|  | Urea + NBPT_1_ | 387.5b | 578.8a | 1590.0bc | 165.9bc | 212.0a | -- |
|  | Urea + NBPT_2_ | 193.6b | 280.2a | 1381.0bc | 306.7ab | 252.8a | -- |
|  | Urea + MIC | 1364.0ab | 842.1a | 4193.0a | 505.1a | 1034.1a | -- |
|  | PCU | 120.7b | 126.1a | 864.1bc | 63.7bc | 210.0a | -- |
|  | P-value | 0.0058 | 0.0934 | 0.00007 | 0.0193 | 0.1752 | -- |
|  |  |  |  |  |  |  |  |
| Jackson-2014 | Zero N | 3.4b | 37.0a | 28.0c | 16.8c | 14.0c | 33.6b |
|  | AN | 25.8b | 81.8a | 70.6c | 31.4c | 25.2c | 84.0b |
|  | Urea | 470.4a | 420.0a | 154.6c | 95.2c | 28.0c | 53.8b |
|  | Urea + NBPT_1_ | 87.4b | 395.4a | 1019.0a | 552.2a | 296.8a | 520.8ab |
|  | Urea + NBPT_2_ | 65.0b | 445.8a | 720.2ab | 305.8b | 232.4ab | 688.8a |
|  | Urea + MIC | 647.4a | 490.6a | 403.2bc | 201.6bc | 78.4bc | 117.6b |
|  | PCU | 134.4b | 451.4a | 711.2ab | 238.6bc | 226.8ab | 260.9ab |
|  | P-value | 0.0028 | 0.0665 | 0.0332 | 0.0147 | 0.0221 | 0.2465 |
|  |  |  |  |  |  |  |  |
| Jackson-2015 | Zero N | 4.5b | 5.6b | 4.5b | 4.5d | 5.6d | 21.3a |
|  | AN | 78.4b | 84.0b | 62.7b | 50.4cd | 32.5cd | 116.5a |
|  | Urea | 1509.0a | 2176.0a | 1083.0a | 630.6a | 397.6a | 313.6a |
|  | Urea + NBPT_1_ | 54.9b | 243.0b | 367.4b | 365.1ab | 234.1ab | 296.8a |
|  | Urea + NBPT_2_ | 51.5b | 212.8b | 390.8b | 397.6ab | 238.6ab | 282.2a |
|  | Urea + MIC | 1195.0a | 1757.0a | 1066.0a | 288.9bcd | 152.3bcd | 184.8a |
|  | PCU | 41.4b | 175.8b | 194.9b | 227.4bcd | 199.4bc | 437.9a |
|  | Urea + NBPT_3_ | 65.0b | 229.6b | 305.8b | 322.6bc | 227.4ab | 235.2a |
|  | P-value | 0.0001 | 0.0016 | 0.0486 | 0.0370 | 0.0317 | 0.1292 |

**Table 1A.** Effects of urea treated with urease inhibitors or polymer coating on ammonia volatilization loss at Milan and Jackson during 2013-2015. Means in a column within each site-year followed by the same letter are not significantly different at *P* = 0.05 according to the Fisher’s protected LSD. AN: Ammonium nitrate; NBPT_1_: N-(n-butyl) thiophosphoric triamide 20%; NBPT_2_: N-(n-butyl) thiophosphoric triamide 26.7%; NBPT_3_: N-(n-butyl) thiophosphoric triamide 30%; MIC: maleic-itaconic copolymer; PCU: Polymer coated urea.

| **Site-year**  **Treatment** | **Grain N conc.** | **Grain N removal** | **N agronomic use efficiency** |
| --- | --- | --- | --- |
|  | **g kg^-1^** | **kg ha^-1^** | **%** |
|  |  |  |  |
| Milan-2013 |  |  |  |
| Zero N | 8.5c | 43.9f | -- |
| AN | 9.9a | 109.3a | 53.1a |
| Urea | 8.7c | 61.4e | 13.9d |
| Urea + NBPT_1_ | 9.1bc | 78.9cd | 28.4bc |
| Urea + NBPT_2_ | 9.2bc | 84.5bc | 32.8b |
| Urea + MIC | 8.7bc | 66.9de | 18.6cd |
| PCU | 9.4ab | 93.4b | 40.2b |
| P-value | 0.0077 | <0.0001 | <0.0001 |
|  |  |  |  |
| Jackson-2014 |  |  |  |
| Zero N | 8.5d | 79.4e | -- |
| AN | 10.4a | 191.4a | 90.9a |
| Urea | 8.6cd | 110.1d | 25.0d |
| Urea + NBPT_1_ | 9.7ab | 158.7b | 64.4b |
| Urea + NBPT_2_ | 9.5b | 139.6bc | 48.9bc |
| Urea + MIC | 9.3bc | 127.8cd | 39.3cd |
| PCU | 9.7ab | 158.4b | 64.1b |
| P-value | 0.0008 | <0.0001 | <0.0001 |
|  |  |  |  |
| Jackson-2015 |  |  |  |
| Zero N | 11.6c | 44.1d | -- |
| AN | 13.6a | 117.2a | 59.3a |
| Urea | 11.7c | 74.7c | 24.9c |
| Urea + NBPT_1_ | 12.5bc | 94.0b | 40.5b |
| Urea + NBPT_2_ | 12.6bc | 92.6b | 39.3b |
| Urea + MIC | 12.0bc | 96.3b | 26.6c |
| PCU | 12.9ab | 76.9c | 56.1a |
| Urea + NBPT_3_ | 12.2bc | 113.2a | 42.3b |
| P-value | 0.0436 | <0.0001 | <0.0001 |

**Table 2A.** Effects of urea treated with inhibitors or polymer coating on grain N concentration, grain N removal, and N agronomic use efficiency at Milan and Jackson during 2013-2015.Means in a column within each site-year followed by the same letter are not significantly different at *P* = 0.05 according to the Fisher’s protected LSD. AN: Ammonium nitrate; NBPT_1_: N-(n-butyl) thiophosphoric triamide 20%; NBPT_2_: N-(n-butyl) thiophosphoric triamide 26.7%; NBPT_3_: N-(n-butyl) thiophosphoric triamide 30%; MIC: maleic-itaconic copolymer; PCU: Polymer coated urea.

| Treatment | 2013 | | 2014 | | 2015 | |
| --- | --- | --- | --- | --- | --- | --- |
|  | Milan | SF | Jackson | SF | Jackson | SF |
|  | g kg^-1^ | g kg^-1^ | g kg^-1^ | g kg^-1^ | g kg^-1^ | g kg^-1^ |
|  |  |  |  |  |  |  |
| N source |  |  |  |  |  |  |
| Zero N | 158ab | 195a | 137cde | 189c | 165a | 165a |
| AN | 160a | 197a | 143a | 206a | 165a | 149b |
| Urea | 146d | 192a | 136de | 199ab | 159c | 155ab |
| Urea + NBPT_1_ | 149d | 195a | 139bc | 201ab | 160c | 160ab |
| Urea + NBPT_2_ | 152bcd | 187a | 140b | 200ab | 164a | 157ab |
| Urea + MIC | 150cd | 194a | 135e | 193bc | 161bc | 163ab |
| PCU | 156abc | 197a | 139bc | 201ab | 161bc | 165a |
| Urea + NBPT_3_ | -- | -- | -- | -- | 163ab | 156ab |
|  |  |  |  |  |  |  |
| N rate (kg N ha^-1^) |  |  |  |  |  |  |
| 123 | 152a | 193a | 137a | 197a | 162a | 159a |
| 168 | 154a | 196a | 139a | 200a | 162a | 159a |
|  |  |  |  |  |  |  |
| N source | 0.0009 | 0.5873 | 0.0001 | 0.0328 | 0.0015 | 0.2706 |
| N rate | 0.2806 | 0.4481 | 0.1339 | 0.1630 | 0.1955 | 0.8951 |
| N rate × N source | 0.6912 | 0.1158 | 0.3927 | 0.6976 | 0.4299 | 0.7890 |

**Table 3A.** Effects of urea treated with urea inhibitors or polymer coating on grain moisture at Milan, Jackson, and Springfield during 2013-2015. SF: Springfield. Part of the yield data were cited from a University of Tennessee extension paper (Savoy et al., 2016). Means in a column within the treatments of N sources or N rates followed by the same letter are not significantly different at *P* = 0.05 according to the protected LSD. AN: Ammonium nitrate; NBPT_1_: N-(n-butyl) thiophosphoric triamide 20%; NBPT_2_: N-(n-butyl) thiophosphoric triamide 26.7%; NBPT_3_: N-(n-butyl) thiophosphoric triamide 30%; MIC: maleic-itaconic copolymer; PCU: Polymer coated urea.

**Figure 1A.** The monthly average air temperature and rainfall at Springfield, Milan, and Jackson during the growing season of 2013 to 2015. SF: Springfield. Seasonal rainfall: SF 2013: 1081 mm, SF 2014: 812 mm, SF 2015: 776 mm, Milan 2013: 1171 mm, Jackson 2014: 1069 mm, and Jackson 2015: 803 mm.
